# Supplementary material for: Content-rich biological network constructed by mining PubMed abstracts
Source: BMC Bioinformatics. 2004 Oct 8;5:147. doi: 10.1186/1471-2105-5-147 (PMC528731; doi:10.1186/1471-2105-5-147)
Supplement: Additional File 2 — The original results of the above study (non-essential files are deleted to keep the file size under the limit set by BMC bioinformatics). [file 1471-2105-5-147-S2.bz2 › chilibotAdditionalFile2/dip05/177ID9417082E615/html/PLAUR_TYK2.html]

 


 **PLAUR** and **TYK2** 
  
Found 4 abstracts in PubMed, retrieved 4.  
 

 What does Google say? 
 PDF only 
| .edu only 

---

**Interactive relationship** (e.g. stimulation, inhibition, etc)

**Stimulatory relationship**- In human vascular smooth muscle cell VSMC uPA stimulates migration via the uPA receptor  [ **PLAUR** ]  uPAR  [ **PLAUR** ]  signalling complex containing the Janus kinase  **Tyk2**  and phosphatidylinositol 3 kinase PI3 K.  Ref: 12719789 Thromb Haemost, 2003
**Neutral relationship**- By using co immunoprecipitation techniques and in vitro kinase assays, the uPAR  [ **PLAUR** ]  associated proteins were identified as Janus and Src non receptor protein tyrosine kinases Jak1,  **Tyk2** , and p59, p53 56, p53 59, and p55.  Ref: 9417082 J Biol Chem, 1998

**Non-interactive relationship** (e.g. studied together, co-existance, homology, etc.)

- Migration experiments with double immunofluorescence staining revealed polarization of uPAR  [ **PLAUR** ] , and colocalization with Jak1 and  **Tyk2**  to the leading edge of the migrating cells.  Ref: 9417082 J Biol Chem, 1998
- Our results provide evidence that the small GTPases RhoA and Rac1, together with Rho kinase, are necessary to mediate the uPA uPAR  [ **PLAUR** ]  directed migration via the  **Tyk2**  PI3 K signalling complex in human VSMC.  Ref: 12719789 Thromb Haemost, 2003
- We report that active GTP bound forms of small GTPases RhoA and Rac1, but not Cdc42, are directly associated with  **Tyk2**  and PI3 K in an uPA uPAR  [ **PLAUR** ]  dependent fashion.  Ref: 12719789 Thromb Haemost, 2003
